# The Viral Threat in Cotton: How New and Emerging Technologies Accelerate Virus Identification and Virus Resistance Breeding

**DOI:** 10.3389/fpls.2022.851939

**Published:** 2022-04-05

**Authors:** Roberto Tarazi, Maite F. S. Vaslin

**Affiliations:** ^1^Plant Molecular Virology Laboratory, Department of Virology, Microbiology Institute, Universidade Federal do Rio de Janeiro (UFRJ), Rio de Janeiro, Brazil; ^2^Programa de Pós-graduação em Biotecnologia e Bioprocessos da UFRJ, Rio de Janeiro, Brazil

**Keywords:** cotton, cotton viruses, gossypium (cotton), breeding, begomovirus disease complex, *Solemoviridae*, virus diagnosis

## Abstract

Cotton (*Gossypium* spp. L., *Malvaceae*) is the world’s largest source of natural fibers. Virus outbreaks are fast and economically devasting regarding cotton. Identifying new viruses is challenging as virus symptoms usually mimic nutrient deficiency, insect damage, and auxin herbicide injury. Traditional viral identification methods are costly and time-consuming. Developing new resistant cotton lines to face viral threats has been slow until the recent use of molecular virology, genomics, new breeding techniques (NBT), remote sensing, and artificial intelligence (AI). This perspective article demonstrates rapid, sensitive, and cheap technologies to identify viral diseases and propose their use for virus resistance breeding.

## Introduction

*Gossypium* spp. L. (*Malvaceae*) is the largest source of natural fibers in the world. The widely cultivated cotton cultivars are allotetraploid species. More than 90% of the annual cotton crop worldwide is *Gossypium hirsutum* (AD_1_), Upland or American cotton, and less than 10% is *Gossypium barbadense* (AD_2_), extra-long-staple, or Pima cotton (USDA, 2021). Global cotton harvested area in 2021 was 32 million hectares, and the production was 24.1 million metric tons. India, China, the United States, Brazil, and Pakistan are the world’s top five cotton producers (ICAC, 2021).

Cotton breeding’s goal is to deliver the most productive varieties in addition to a high-quality fiber standard (Hu et al., 2019; Wang et al., 2019; Yang et al., 2020). Breeding a new cotton variety may take near a decade until its commercial launch. Furthermore, additional time is required for commercialization when staking a new market-regulated transgene in the variety’s genome (Tarazi et al., 2020).

High-yield and fiber quality prioritization and the adoption of economic models aid breeding companies in selecting the traits, minimizing the investment risk, and guaranteeing variety’s commercial longevity (Louwaars et al., 2012; Ceccarelli, 2015). Despite the relevance of prioritization and economic models, companies do not consider pathogens’ mutation rate and spread of viral vectors’ speed, neglecting new or local pathogens that have the potential for pandemics (Jones, 2009). Viruses’ mutation rates, reaching up to a million times higher than their hosts, are associated with viruses’ replication speed and favor the emergence of hundreds of new virus strains at each infection (Duffy, 2018). Pathogen mutation consistently exceeds the breeding selection speed causing pathogen outbreaks and high economic loss (Schenke and Cai, 2020).

In virus-resistant cotton varieties, viral replication is suppressed or null; nevertheless, viruses replicate in high titers in virus-tolerant cotton varieties (Lapidot and Friedmann, 2002), weeds, or reservoir plants growing near the cotton plantation (Duffus, 1971). The perenniality of weeds or reservoir plants in the cotton fields favors viral high mutations accumulation and the emergence of new virus strains capable of breaking the viral resistance of cotton varieties. Cotton leafroll dwarf virus (CLRDV) in weeds and overwintering cotton in the United States shows an example of virus maintenance during the counter season generating a disease outbreak during the growing season (Sedhain et al., 2021). In addition, climate change is accelerating even more pathogen and vector spread and establishment (Jones, 2009; Chaloner et al., 2021).

A fast viral identification in the field using molecular virus detecting techniques (MVDT), digital disease assessment and phenotyping (DDAP), artificial intelligence (AI), and biotechnological tools may accelerate and enhance the breeding of virus-resistant cotton and help sustain the cotton industry.

## The Viral Threat in Cotton

The known viruses that infect cotton worldwide belong majority to the *Solemoviridae* (former: *Luteoviridae*; ICTV, 2021) and *Geminiviridae* families, whose main vectors are aphids and whiteflies, respectively (Ziegler-Graff, 2020; Wang and Blanc, 2021). The cotton leaf roll dwarf virus (CLRDV, Genus: *Polerovirus*; Family: *Solemoviridae*), a positive sense (+) ssRNA virus, causes the Cotton Blue Disease (CBD) that affects cotton crops in South America and Africa (Distéfano et al., 2010; Parkash et al., 2021). *Aphis gossypii* (Glover; Hemiptera: *Aphididae*), one of the most polyphagous aphid species and worldwide-distributed, hosts over 40 viruses (Quan et al., 2019) and is the exclusive vector of CLRDV (Michelotto and Busoli, 2007; Ramos-sobrinho et al., 2021).

Since 2017, CBD has spread in important cotton grower states in the United States (Avelar et al., 2019; Parkash et al., 2021; Ramos-sobrinho et al., 2021). Curiously, due to very rigorous winters, *A. gossypii* does not impose high pressure in the United States cotton fields (Carletto et al., 2009), suggesting two hypotheses: first, CLRDV may have adapted to a new viral vector and second, even in low aphid pressure, the insects can perpetuate disease spread and maintenance. However, as the viruses circulate all the crop areas, new severe strains can arise anytime.

CLRDV susceptible cotton cultivars may lose all their yield in the presence of the virus. At the end 1990s, the Brazilian cotton industry nearly collapsed due to CDB (Galbieri et al., 2017). The null or reduced yield imposed by CLRDV obligates Brazilian producers to seek resistant varieties (Hoffmann et al., 2019). Today, almost all cotton varieties grown in Brazil are CBD resistant (da Silva et al., 2015). Moreover, the control of *A. gossypii* by insecticides is crucial for cotton growth and involves high costs and environmental contamination (Allen et al., 2018).

In 2006, new CLRDV strains able to break variety’s CBD resistance were detected in Brazil and Argentina and were responsible for a “new” CBD disease, namely, Atypical Cotton Blue Disease (ACBD; da Silva et al., 2015). The known vector continues to be *A. gossypii*, and losses associated with ACBD are significantly lower when compared to CDB (Hoffmann et al., 2019). Between 2012 and 2021, India (Mukherjee et al., 2012), Thailand (Sharman et al., 2015), Timor-Leste (Ray et al., 2016), Sudan (Kumari et al., 2020), Uzbekistan (Moukahel et al., 2021), and Australia (Davis et al., 2021) have reported CLRDV.

Although *Polerovirus* exists in some Asian countries, cotton’s primary viruses in Asia are single-stranded DNA viruses from the *Begomovirus* genus, *Geminiviridae* family. The *Begomovirus* vector is the whitefly *Bemisia tabaci* (Gennadius; Hemiptera: *Aleyrodidae*), a “cryptic species complex,” which is one of the most agriculturally important pests worldwide (Wang and Blanc, 2021). Cotton Leaf Curl Disease (CLCuD) severely impacts cotton production in India and Pakistan. Since the identification of CLCuD in the 1960s, CLCuD studies show the association of *Begomovirus* species, mainly cotton leaf curl Multan virus (CLCuMuV) or cotton leaf curl Kokhran virus (CLCuKoV), with the cotton leaf curl Multan betasatellite (CLCuMuB; Zubair et al., 2017).

The 1992–1993 CLCuD epidemic in Pakistan caused economic losses calculated in one billion US dollars (Briddon and Markham, 2000) and is an example of the tremendously deleterious effect of the illness. Even with the continuous deployment of CLCuD tolerant and resistant varieties, virus resistance outbreak continues to impose severe issues in Asian cotton producers’ countries (Zaidi et al., 2020). With climate change accelerating vector and pathogen spread and establishment (Chaloner et al., 2021), *Begomovirus* may collapse the world’s other top three cotton producers (China, the United States, and Brazil) as *B*. *tabaci* is present in their territories.

## The Growing Menace

In the American continent, the cotton chlorotic spot virus is an emerging *Begomovirus* that mimics nutrition deficiency, is hard to diagnose, and proliferates through the Brazillian cotton belt in the Midwest and Northeast regions (de Almeida et al., 2013). Poleroviruses such as the Brazilian cotton anthocyanosis virus (CAV; Costa and Sauer, 1954), CLRDV divergent strains (Silva et al., 2008), and Argentinian cotton leafroll bushy virus (CLRBV; Agrofoglio et al., 2017) are elevating their frequency in the fields. In the United States, the cotton leaf crumple virus (CLCrV; Brown and Nelson, 1984), endemic to the Southwestern United States region and Mexico, is spreading to the West United States region and Guatemala (Idris and Brown, 2004).

African begomoviruses such as the cotton yellow mosaic virus (CYMV) and cotton leaf curl Gezira virus associated with cotton leaf curl Gezira betasatellite and cotton leaf curl Gezira alphasatellite, and African cotton mosaic disease virus is spreading at a quick rate from Western to Central Africa and Indo-Pak subcontinent (Leke et al., 2016).

The cotton bunchy top (CBT), a polerovirus from the Oceania continent, is cotton’s most viral severe disease in Australia. CBT often occurs in patches or on crop edges and is associated with the highest aphid activity areas (Ellis et al., 2013).

Interestingly, inter-specific virus contamination and coinfection are rising among crops worldwide. The tobacco streak virus (TSV, genus *Ilavirus*, family *Bromoviridae*), transmitted by thrips, was reported to infect cotton in specific regions of India and Pakistan (Jagtap et al., 2012). In China, a novel member of the *Polerovirus* genus and the watermelon mosaic virus (WMV, genus: *Potyvirus*, Family: *Potyviridae*) transmitted by aphids are coinfecting cotton plants (Yang et al., 2021). The mentioned examples make cotton virus resistance breeding and real-time monitoring an urgent need.

## Virus Resistance Breeding and New Molecular Virus Detection Technologies

Starting a cotton breeding program for virus resistance needs the correct definition of “resistance.” A host plant is resistant if it can suppress the multiplication of a virus and consequently suppress disease symptoms, regardless of the resistance mechanism. On the other hand, tolerance is a unique instance where the host expresses minor or mild disease symptoms in response to virus infection but supports virus multiplication (Cooper and Jones, 1983; Mandadi and Scholthof, 2013). Selecting tolerant lines that produce high-yield and fiber quality rather than resistant ones may lead to viruses replicating in high titers and new virus strains with enhanced virulence and evolvability (Lapidot and Friedmann, 2002).

Virus resistance breeding requires virus identification and screening of cotton plants to identify resistance. Serological and molecular virus detecting techniques (MVDT) are the safest way to confirm a virus species and/or strain and a resistant cotton line. After confirming viral resistance, breeders can plan the crosses and breeding schemes. Moreover, molecular and discovery breeders can initiate molecular marker discovery for resistant genes and molecular breeding strategies with the knowledge of contrasting resistant and susceptible cotton lines.

Nowadays, molecular virus detecting techniques, such as high-throughput sequencing (HTS), allow the detection of all viruses present in a plant (virome), including undescribed new viruses (Sharma et al., 2021). However, finding a direct association between the disease and a particular virus among those detected in the infected plant is necessary to fulfill Koch’s postulates.

Virome information is essential for breeding and molecular breeding schemes to understand the availability and type of viral-resistant genes needed for elite line introgression. Developing a new resistant elite variety can be fast if a known virus causes the symptoms and resistant germplasm and molecular markers are available. When dealing with a new virus or coinfection, developing a new resistant elite variety will demand germplasm and gene prospection, resources, and time.

After completing the virus sequencing step, it is possible to develop new sensitive virus detection techniques and replace costly qRT-PCR-based diagnosis testing systems (Srivastava et al., 2020). Some accurate and ultrasensitive nucleic acid-based testing (NAT) tools in development for multiplexed virus infection capable of replacing qRT-PCR are recombinase polymerase amplification (RPA; Kim et al., 2018), loop-mediated isothermal amplification (LAMP; Selvaraj et al., 2019), and clustered regularly interspaced short palindromic repeats (CRISPR; Kellner et al., 2019). Viruses identification by NAT multiplex systems will be fundamental to identifying multiple resistant germplasm, accelerating host molecular marker discovery for resistance traits, and developing resistant varieties.

Among CRISPR-NAT tools, CAS9 has shown to be an ultrasensitive pathogen detection method. Converting the developing NAT to point-of-care testing (POCT) will drastically reduce costs and accelerate viral identification due to on-site identification and easy handling (Srivastava et al., 2020). CRISPR/Cas9-mediated lateral flow nucleic acid assay (CASLFA; Wang et al., 2020) and Editor-Linked Uniform Detection Assay (FELUDA; Azhar et al., 2021) are new POCT tools optimized for fast and easy *in situ* screening of human viruses using paper strips. The development of such POCT tools for plant viruses will enable a large-scale, fast, and robust detection of virus on-site, aiding in selecting virus-resistant cotton lines without waiting for symptoms.

MVDT are so fast and sensitive that CLRDV is detectable in susceptible cotton plants 24 h after inoculation and 2–5 days in systemic leaves after aphid infection, respectively (Fausto et al., 2017). MVDT contrast with slow and subjective symptom-based-pathological trials that take over 90 days and lead to a high probability of selecting tolerant lines. In other words, the development of new MVDT for circulating cotton virus will accelerate resistance selection and aid in developing molecular markers for molecular breeding strategies.

## Digital Disease Assessment and Phenotyping

**Figure 1:**
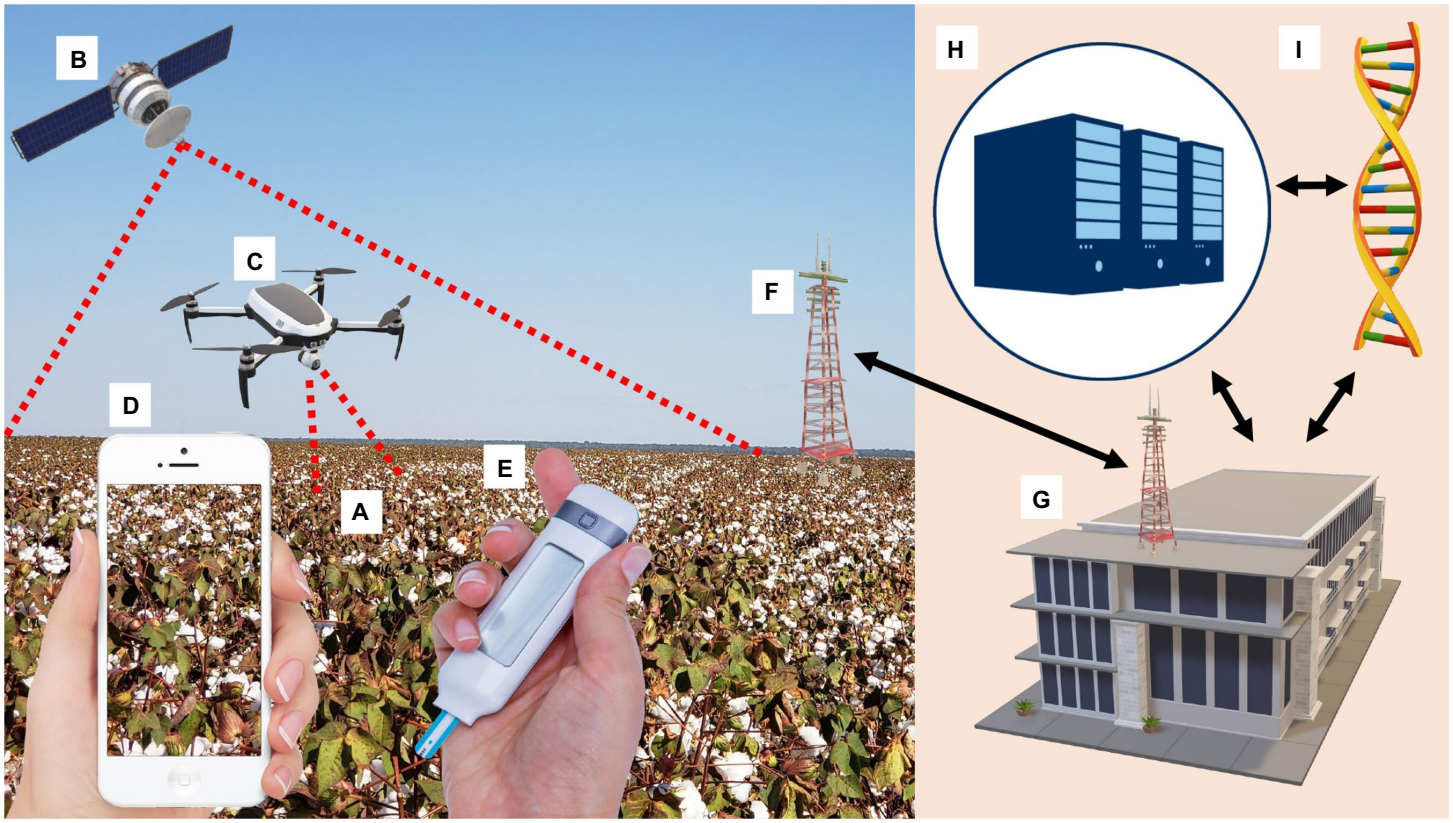
Artificial Intelligence (AI) and Molecular Virus Detecting Techniques (MVDT) calibrating Digital Disease Assessment and Phenotyping (DDAP) to determine whether a cotton plant has nutrition deficiencies, insect damage, auxin herbicide injury, or symptoms resulting from viral infection. **(A)** Symptomatic cotton field; **(B)** Satellite provides land monitoring on a large scale and captures spectral signature anomalies caused by viruses; **(C)** Unmanned Aerial Vehicles (UAVs or “drones”) analyse individual plants and rows from plots; **(D)** Smartphone digital apps capture, analyze, and diagnose annotated pathologies with the help of AI; **(E)** Point-of-care testing (POCT), an MVDT identifies viruses on-site and validates DDAP data; **(F)** 5G wireless technology integrates DDAP and MVDT in the field and laboratory; **(G)** Laboratory hosts MVDT and server for Deep Learning (DL) image processing; **(H)** DL servers gather information from MVDT and DDAP, gives a solution for the symptomatic cotton field, and calibrates DDAP to recognize viral infection autonomously; and **(I)** MVDT laboratory can receive material from the field and gives the results of the presence or absence of viruses and inputs data for DL process.

Several available technologies permit digital disease assessment and phenotyping (DDAP) and may reduce operational time, costs, breeding score subjectivity, and increases the genetic gain (ΔG) by increasing selection intensity (*i*) and selection accuracy (*r*), parameters of the “breeder’s equation” $\Delta G=\;\sigma_{a}\;i\;r/L$ (Cobb et al., 2019). With the help of artificial intelligence (AI) and MVDT calibration, DDAP can determine whether a cotton plant has nutrition deficiencies, insect damage, auxin herbicide injury, or symptoms resulting from viral infection (Figure 1).

Viral diseases change healthy vegetation’s spectral signature characterized by a specific reflectance peak in the near-infrared (NIR; 800–900 nm) and lower reflectance values in the red region (650–750 nm; Mahlein, 2016). With the help of AI and high-resolution images, pre-symptomatic disease detection can be inferred by multi- and narrowband visible to shortwave infrared (SWIR; 400–2,500 nm; Jung et al., 2021).

The use of DDAP reduces MVDT costs by narrowing the number of potentially resistant plants tested for viral presence during breeding trials and germplasm screening. DDAP in cotton breeding covers large scales and multiple sites using satellites, downscaling to row, or individual plant analyses from plots in a field using drones until reaching small spaces such as greenhouses or laboratories using smartphones.

Smartphones are accessible, easy-to-manage image analysis platforms that can aid researchers and breeders in diagnosing diseases (Silva et al., 2021). Smartphone digital cameras use RGB (red, green, and blue), and apps capture, analyze, and diagnose annotated pathologies with the help of AI (Mahlein, 2016).

Unmanned Aerial Vehicles (UAVs or “drones”) can detect viral diseases with decreased dependence on weather conditions (Silva et al., 2021). Swarm robotics increases drone data acquisition efficiency per hectare in large farms, competing with satellite imagery prices (Schranz et al., 2020).

Satellites provide land monitoring on a large scale from 100’s to 1000’s km^2^ of data and can capture healthy vegetation’s spectral signature and anomalies caused by viruses (Oerke, 2020), such as CLCuD (Ahmed et al., 2013). Using a constellation of multiple satellites, small satellites, or even a mega-constellation of mini-satellites improves revisit time, reducing weather dependency and increasing spectral resolution (Zhang et al., 2020).

5G wireless technology implementation will integrate DDAP and MVDT in the field and laboratory, as it has the theoretical capacity to connect one million devices per square kilometer and deliver speeds faster than 10 Gbps (Tang et al., 2021). The sum of all or some of these technologies with MVDT will enable the virus identification on-site.

## Deep Learning an Efficient AI Imaging Solving Tool

High-throughput phenotyping to help develop virus-resistant varieties is possible through AI, nevertheless, incorrect input biases AI learning, leading to disastrous results (Barbedo, 2018). The correct screening (phenotyping) of resistant lines for the AI learning process must consider the definition of “resistance.” Thus, MVDT can provide the correct input in the AI learning process.

As AI requires large data sets (Big Data), labeling training data, and high processing time, Deep Learning (DL) enables an automatic and hierarchical learning process (Barbedo, 2018; Kamilaris and Prenafeta-Boldú, 2018). DL has shown an efficient AI tool in solving images of several diseases, including cotton leaf disease (Ahmed, 2021). DL belongs to Machine Learning (ML) and refers to artificial neural networks with many layers that automatically determine image features by the network. DL has advantages over ML as it reduces feature engineering in image processing (Liakos et al., 2018; Jung et al., 2021).

## Accelerated Resistance Breeding Using Molecular and Biotechnological Tools

Pathogen-resistant cotton lines appear to have relatively low yields, low fiber quality, and high linkage drag, complicating the development of elite resistant varieties. Implementing integrated cotton molecular breeding strategies (Yang et al., 2020), such as Marker Assisted Selection (MAS), Marker Assisted Introgression (MAI), Multi-parent Advanced Generation Inter-Cross populations (MAGIC), Genome-Wide Association Studies (GWAS), and Genomic Selection (GS), with DDAP and MVDT, will enhance the development of improved yield, fiber quality, and virus-resistant cotton varieties.

Moreover, new breeding techniques (NBT) may be the quickest path to developing elite resistant varieties without the worries of linkage drag (Tarazi et al., 2020). Among the NBT, the CRISPR/Cas system is easy to use and can efficiently generate targeted mutagenesis, conferring molecular immunity against eukaryotic viruses, including cotton DNA geminiviruses (Ali et al., 2016).

In the absence of native resistance traits, transgenic approaches are necessary. Genetically modified (GM) plants expressing the antisense movement protein (*AV2*) and antisense coat protein (*ACP*) genes of CLCuV provide resistance to CLCuD (Sanjaya et al., 2005). Transgenic cotton plants that overexpress miR166 show potential in reducing *Bemisia tabaci* populations and, more importantly, the spread of whitefly-transmitted plant viruses (Wamiq and Khan, 2018).

**Figure 2:**
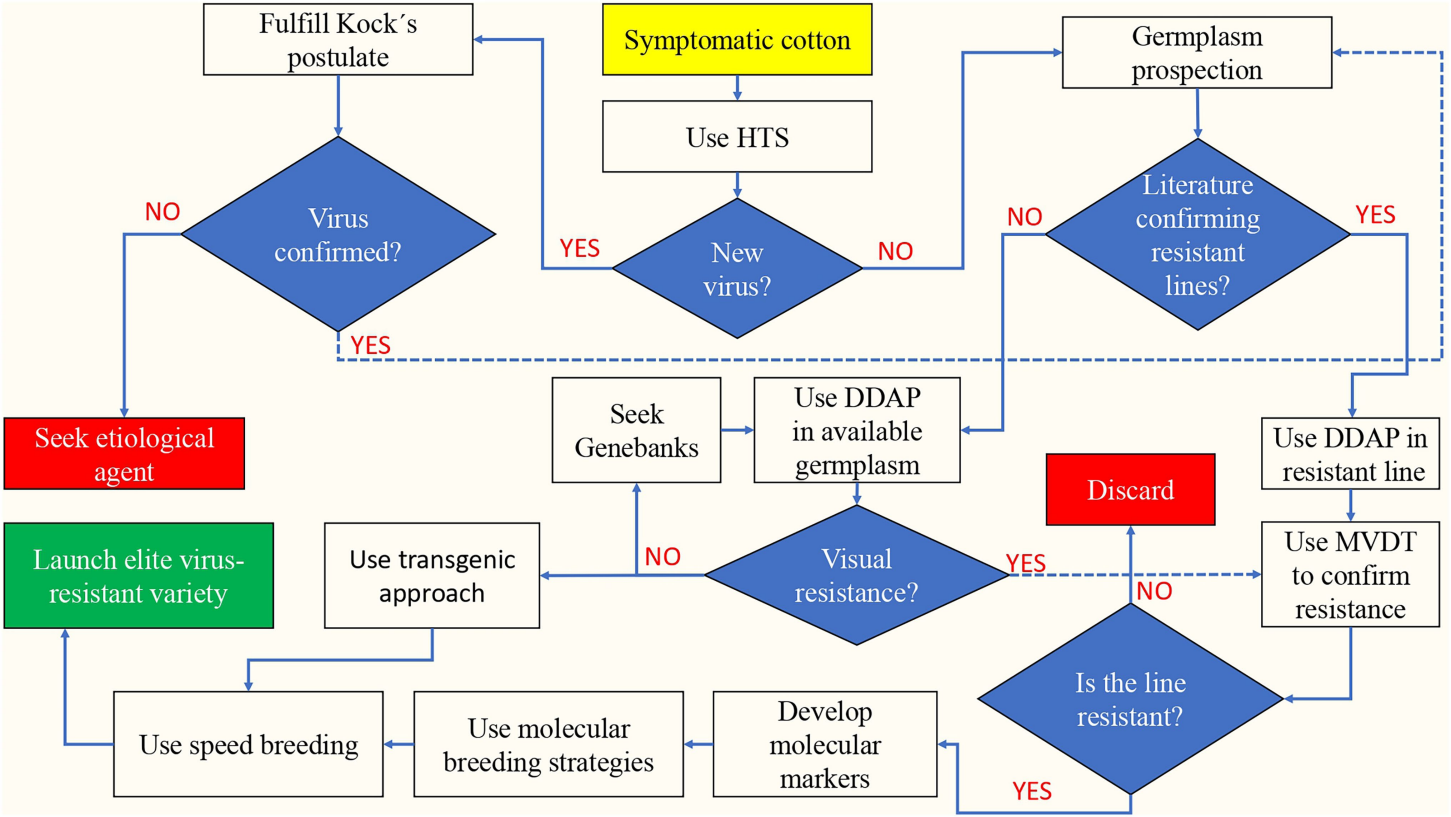
Flowchart to accelerate and enhance the breeding of virus-resistant cotton based on the identification of symptomatic cotton plants in the field. HTS, High-throughput sequencing; MVDT, Molecular virus detecting techniques; and DDAP, Digital disease assessment and phenotyping.

Implementing the Speed Breeding approach (Watson et al., 2018) will accelerate the deployment of viral-resistant cotton varieties to the fields through rapid generation advancement. Figure 2 shows a flowchart to accelerate and enhance the breeding of virus-resistant cotton after discovering virus-like cotton symptomatic plants in the field, using the combination of new recently available DDAP, MVDT, molecular breeding, GM, and Speed Breeding technologies.

## Finding Resistant Germplasm

A significant problem in breeding is finding resistant germplasm for current and emerging viruses. GeneBanks are a solution for finding resistant genetic material (Van Treuren and Van Hintum, 2014). The International Treaty on Plant Genetic Resources for Food and Agriculture (ITPGRFA) aids in the sustainable use of plant genetic resources, stimulating GeneBanks (FAO, 2021).

However, the fair and equitable sharing of the benefits of ITPGRFA creates complicated bureaucratic mechanisms that slow or obliterate resistant material deployment in the face of high viral mutation rates and spread. ITPGRFA needs to be updated to face immediate and devastating viral threats. In the absence of resistant cotton varieties, viruses might knock down the cotton global industry chain that involves approximately 150 countries and indirectly provides food security for around 100 million families.

## Conclusion

The viruses mentioned in this perspective have cost billions of US dollars for the cotton industry over the past decades. The investment needed in MVDT and DDAP that accelerates and enhances virus identification and the breeding of virus-resistant cotton costs less than one year of economic losses caused by a virus outbreak. Besides that, the use of MVDT must be mandatory in virus-resistant breeding programs, as virus-tolerant cotton varieties are viral time bombs that can cause severe economic losses. Thus, combining and improving available MVDT and DDAP seem to be the way to accelerate viral disease identification and deploy resistant cotton varieties to suppress viral outbreaks and help sustain the cotton industry.

## Data Availability Statement

The original contributions presented in the study are included in the article/Supplementary Material, further inquiries can be directed to the corresponding author.

## Author Contributions

RT and MV devised the structure and decided on the content of the paper. RT conducted the literature survey, then both RT and MV jointly wrote the manuscript and contributed to revisions. All authors contributed to the article and approved the submitted version.

## Funding

Part of this research was funded by Fundação Carlos Chagas Filho de Amparo à Pesquisa do Estado do Rio de Janeiro (FAPERJ) project E-26/010.001716/2019.

## Conflict of Interest

The authors declare that the research was conducted in the absence of any commercial or financial relationships that could be construed as a potential conflict of interest.

## Publisher’s Note

All claims expressed in this article are solely those of the authors and do not necessarily represent those of their affiliated organizations, or those of the publisher, the editors and the reviewers. Any product that may be evaluated in this article, or claim that may be made by its manufacturer, is not guaranteed or endorsed by the publisher.
